# Medulloblastoma in China: Clinicopathologic Analyses of SHH, WNT, and Non-SHH/WNT Molecular Subgroups Reveal Different Therapeutic Responses to Adjuvant Chemotherapy

**DOI:** 10.1371/journal.pone.0099490

**Published:** 2014-06-16

**Authors:** Zhen-Yu Zhang, Jian Xu, Yong Ren, Yu Yao, Kay Ka-Wai Li, Ho-Keung Ng, Ying Mao, Liang-Fu Zhou, Ping Zhong

**Affiliations:** 1 Department of Neurosurgery, Huashan Hospital, Fudan University, Shanghai, China; 2 Department of Pathology, Wuhan General Hospital of Guangzhou Command, People's Liberation Army, Wuhan, China; 3 Department of Anatomical and Cellular Pathology, Prince of Wales Hospital, The Chinese University of Hong Kong, Hong Kong, China; University of Navarra, Spain

## Abstract

Medulloblastoma (MB) is one of the most common primary central nervous system tumors in children. Data is lacking of a large cohort of medulloblastoma patients in China. Also, our knowledge on the sensitivity of different molecular subgroups of MB to adjuvant radiation therapy (RT) or chemotherapy (CHT) is still limited. The authors performed a retrospective study of 173 medulloblastoma patients treated at two institutions from 2002 to 2011. Formalin-fixed paraffin embedded (FFPE) tissues were available in all the cases and sections were stained to classify histological and molecular subgroups. Univariate and multivariate analyses were used to investigate prognostic factors. Of 173 patients, there were 118 children and 55 adults, 112 males and 61 females. Estimated 5-year overall survival (OS) rates for all patients, children and adults were 52%, 48% and 63%, respectively. After multivariate analysis, postoperative primary radiation therapy (RT) and chemotherapy (CHT) were revealed as favorable prognostic factors influencing OS and EFS. Postoperative primary chemotherapy (CHT) was found significantly improving the survival of children (p<0.001) while it was not a significant prognostic factor for adult patients. Moreover, patients in WNT subtype had better OS (p = 0.028) than others (SHH and Non-SHH/WNT subtypes) given postoperative adjuvant therapies. Postoperative primary RT was found to be a strong prognostic factor influencing the survival in all histological and molecular subgroups (p<0.001). Postoperative primary CHT was found significantly to influence the survival of classic medulloblastoma (CMB) (OS p<0.001, EFS p<0.001), SHH subgroup (OS p = 0.020, EFS p = 0.049) and WNT subgroup (OS p = 0.003, EFS p = 0.016) but not in desmoplastic/nodular medulloblastoma (DMB) (OS p = 0.361, EFS p = 0.834) and Non-SHH/WNT subgroup (OS p = 0.127, EFS p = 0.055). Our study showed postoperative primary CHT significantly influence the survival of CMB, SHH subgroup and WNT subgroup but not in DMB and Non-SHH/WNT subgroup of MB.

## Introduction

Medulloblastoma is one of the most common primary central nervous system tumors in children, with an estimated incidence of 0.5/100,000 children [1], [2]. In contrast, medulloblastoma represents a rare tumor in adults and comprises less than 1% of adult primary brain neoplasms [3]. Current managements of medulloblastoma are mainly based on the results of several prospective studies initiated in North America and Europe [4]. For children, surgical resection followed by craniospinal irradiation (CSI) and chemotherapy (CHT) is the typical treatment modality [4]–[7]. Although there lack prospective clinical trials confirming the benefit of CHT in treating adult patients, there is a trend towards a combined treatment strategies including CHT for adults[8].

Recent progress on medulloblastoma gene expression profiling demonstrated the disease is heterogeneous and consists of different molecular subgroups: SHH, WNT and Non-SHH/WNT medulloblastoma [9]–[12]. These molecular subgroups have been demonstrated to differ in demographic, genetic and clinical aspects. Of note, patients with WNT medulloblastoma survived significantly longer than patients with other subgroups of medulloblastoma, and there is a consensus that patients in this subgroup may be over treated with the possibilities of reducing current therapies such as RT and CHT [11]. Nevertheless, how to reduce adjuvant therapies in WNT medulloblastoma remains unclear. For instance, is it safe to remove CHT from current treatment modality for WNT medulloblastoma? Since our knowledge on the sensitivity of different molecular subgroups to adjuvant RT or CHT is still limited, it may be difficult to answer this question.

In the past decades, clinical and pathological prognostic factors for patients with medulloblastoma have been discussed by numerous reports. However, there are few reports on large cohorts of medulloblastoma patients in China and relevant data are very limited [13]. We performed a retrospective study of medulloblastoma to investigate underlying prognostic factors, and focused on the sensitivity of different molecular subgroups of medulloblastoma to current adjuvant therapies.

## Patients and Methods

### Ethics Statement

This study was approved by the Ethics Committee of Huashan Hospital, Fudan University and Southwest Hospital, Third Military Medical University. Since this is a retrospective study, written informed consent was not given by patients for their clinical records to be used in this study. However, clinical records of all patients in this study were anonymized and de-identified prior to analysis. Ethics Committee of Huashan Hospital, Fudan University and Southwest Hospital, Third Military Medical University had waived the need for written informed consent from the patients.

### Patients and clinical data

A total of 173 medulloblastoma patients pathologically diagnosed in Huashan Hospital (Shanghai, China) and Southwest Hospital (Chongqing, China) between January 2002 and October 2011 were included in this study. Clinical data were collected from medical charts and follow-up data were collected by telephone interviews or clinic follow-ups. These data included age, sex, neurological symptoms, symptom duration, neuroimaging findings, extent of resection, CSF shunting, postoperative primary radiation therapy (RT), postoperative primary CHT and patients' clinical outcomes.

### Classifications of histological and molecular subgroups

Formalin-fixed paraffin embedded (FFPE) tissues from primary tumor resection were available in all the cases. Haematoxylin & eosin (H&E) stained sections of tumors were reviewed and classified according to the 2007 WHO classification of tumors of central nervous system [14]. One histological subtype is medulloblastoma with extensive nodularity (MBEN), which is closely related to desmoplastic/nodular medulloblastoma (DMB) [14]. There were only 4 specimens classified as MBEN in our series and we added the 4 cases into DMB. Thus, there were 3 histological subtypes in our study: classic medulloblastoma (CMB), DMB and large-cell/anaplastic medulloblastoma (AMB). Immunohistochemistry for molecular subgroups of medulloblastoma was undertaken and evaluated according to the protocol illustrated by Ellison et al with modifications [9]. SHH subgroups, WNT subgroups and non-SHH/WNT subgroups were categorized by different immunoreactivities to two antibodies: β-catenin (BD #610154; 1∶800; antigen retrieval, citrate buffer 20 min Bond), GAB1 (Abcam #ab27439; 1∶50; antigen retrieval, citrate buffer 20 min Bond) (**Table S1**).

### Statistical analysis

Overall survival(OS)was measured from the date of diagnosis to the date of death or last follow-up. Event-free survival(EFS)was measured from the date of diagnosis to the date of disease recurrence, death, or last follow-up. Survival curves were constructed using Kaplan-Meier methods. Differences in OS and EFS between subgroups of patients were analyzed using log-rank test (univariate analysis). Suitable prognostic factors for multivariate analysis were selected based on the p-value (<0.10) in univariate analysis. These factors were subsequently put into Cox proportional hazards regression model to identify independent prognostic factors (multivariate analysis). Statistical significance was defined as p-value being less than 0.05. Data statistics were performed using software IBM SPSS Statistics 19 (IBM Corp., Armonk, NY, USA).

## Results

### Patients' characteristics

There were 118 children (3≤age<16 years) and 55 adults (age≥16 years) with a children to adults ratio of 2.1∶1. The median age at diagnosis was 12 years (range, 3–45 years). A male preponderance was observed in our cohort: 112 (65%) were male and 61 (35%) were female with a male to female ratio of 1.8∶1. The most common symptoms were headache (69%) and nausea/vomiting (55%), which were associated with increased intracranial pressure. Cerebellar dysfunctional symptoms such as ataxia (40%) and diplopia (6%) were also common. Duration of symptoms varied from 0.5 day to 1.5 years and median duration of symptoms was 1.1 month. Except for those patients with missing information (26 cases), 63 (43%) patients had tumor infiltrating brainstem or IV ventricle floor. Radiological examinations revealed hydrocephalus in 111 patients (70%), with missing data in 15 cases (**Table 1**).

**Table 1:** Univariate analysis of prognostic factors for OS and EFS in medulloblastoma patients (n = 173).

Factors	No. of cases	5-year OS(%)	p-value	5-year EFS(%)	p-value
Sex					
Male	112	49.8	0.216	43.2	0.384
Female	61	55.5		48.6	
Age					
Children	118	47.6	0.090	41.9	**0.047**
Adult	55	62.7		51.7	
Location					
Midline	152	50.2	0.219	43.7	0.228
Lateral	21	65.3		54.9	
Involvement^a^					
Yes	63	43.3	**0.002**	35.2	**0.001**
No	84	63.8		55.2	
Unknown	26				
Tumor Size					
<3 cm	110	56.8	0.314	47.7	0.444
≥3 cm	37	44.2		41.8	
Unknown	26				
Extent of resection					
Gross total	114	60.9	**0.002**	53.9	**<0.001**
Subtotal	52	42.7		33.0	
Unknown	7				
CSF Shunting					
Yes	53	45.8	0.074	41.1	0.055
No	103	56.0		47.5	
Unknown	17				
RT^b^					
Yes	139	58.4	**<0.001**	49.3	**<0.001**
No	34	8.0		8.0	
CHT^c^					
Yes	98	62.1	**<0.001**	51.2	**0.001**
No	75	38.9		38.9	
Institution					
A	153	53.3	0.184	46.9	0.238
B	20	40.0		40.0	

a involving brain stem or IV ventricle floor by the tumor.

b indicating postoperative primary radiation therapy.

c indicating postoperative primary chemotherapy.

Institution A =  Huashan Hospital.

Institution B = Southwest Hospital.

### Treatment characteristics

All patients underwent tumor resection: 114 patients (69%) had gross total resection, 36 patients (31%) had subtotal resection and the extent of resection in 7 cases could not be evaluated based on our data. Perioperative CSF shunting was performed in 53 patients (34%) with missing data in 17 patients. In the entire cohort, 139 patients (80%) received postoperative primary RT and 98 patients (57%) received postoperative primary CHT (**Table 1**). There were 94 patients (54%) who underwent postoperative primary RT followed by sequential CHT, 47 patients (27%) who received only postoperative primary RT, 7 patients (4%) who received only postoperative primary CHT and 25 (15%) patients who underwent neither postoperative primary RT nor CHT.

### Survival analysis for prognostic factors

The median follow-up time for all patients was 38.0 months with a range of 0.1 to 118.9 months. Estimated 5-year overall survival rates for all patients, children and adults were 52%, 48% and 63%, respectively. Estimated 5-year event-free survival rates for all patients, children and adults were 45%, 42% and 52%, respectively (**Figure S1**). Univariate analysis of prognostic factors for the entire cohort revealed that absence of brainstem or IV ventricle floor involvement by the tumor, gross total resection of the tumor, reception of postoperative primary RT and CHT were favorable factors significantly influencing OS and EFS (**Table 1**). The p-values of other two parameters (age and CSF shunting) were less than 0.10. Therefore, the 6 clinical parameters (age, CSF Shunting, involvement of brain stem or IV ventricle floor, extent of resection, RT and CHT) were put into multivariate analysis, in which postoperative primary RT and CHT were identified as independent prognostic factors significantly impacting OS and EFS (**Table 2**). The influence of postoperative primary adjuvant therapies to the survival of children and adults with medulloblastoma was further studied by univariate analysis (**Table S2**). Postoperative RT was a favorable prognostic factor for both children and adults (p<0.001). Postoperative primary CHT significantly improved the survival of children (OS p = 0.001, EFS p = 0.003) while it was not a significant prognostic factor for adult patients.

**Table 2:** Multivariate analysis of prognostic factors for OS and EFS in patients with medulloblastoma (n = 173).

Factors	OS		EFS	
	OR(95%CI)	p-value	OR(95%CI)	p-value
Age (Children vs. Adults)	0.69(0.38–1.26)	0.229	0.73(0.42–1.25)	0.251
CSF Shunting(Yes vs. No)	0.71(0.42–1.20)	0.202	0.73(0.45–1.20)	0.201
Involvement^a^ (Yes vs. No)	0.60(0.29–1.22)	0.157	0.70(0.36–1.40)	0.306
Complete resection (Yes vs. No)	1.23(0.58–2.58)	0.590	1.56(0.78–3.10)	0.208
RT^b^ (Yes vs. No)	8.18(4.52–14.80)	**<0.001**	6.74(3.83–11.87)	**<0.001**
CHT^c^ (Yes vs. No)	1.75(1.04–2.96)	**0.036**	1.63(1.00–2.65)	**0.050**

OR: odds ratio, CI: confidence interval.

a involving brain stem or IV ventricle floor by the tumor.

b indicating postoperative primary radiation therapy.

c indicating postoperative primary chemotherapy.

### Survival analysis for histological and molecular subgroups

**Figure 1:**
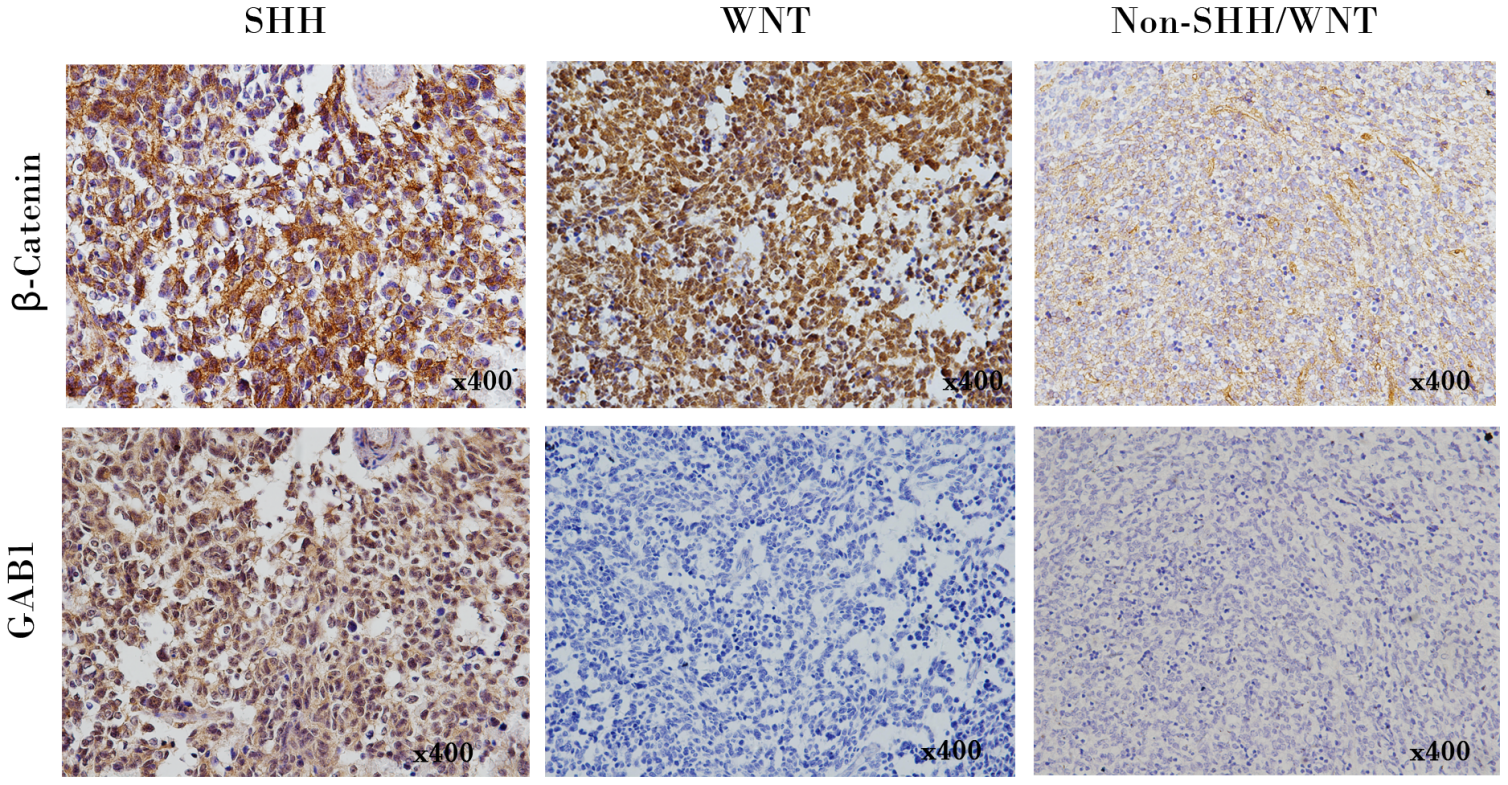
Molecular classification of medulloblastoma by immunohistochemical staining. 173 cases were classified into SHH subgroup (β-Catenin cytoplasmic-positive, GAB1 cytoplasmic-positive), WNT subgroup (β-Catenin nuclear+cytoplasmic-positive, GAB1-negative) and Non-SHH/WNT subgroup (β-Catenin cytoplasmic-positive, GAB1-negative). Representative photomicrographs of each subgroup are shown.

There were 119 CMB, 45 DMB and 9 AMB according to WHO classification. Univariate analysis for OS and EFS between the three histological groups did not find significant differences (**Table 3**). Immunohistochemical staining classified 173 cases into three molecular subgroups (**Figure 1**): SHH subgroup (40 cases), WNT subgroup (37 cases) and Non-SHH/WNT subgroup (96 cases). Univariate analysis for OS and EFS between the three molecular groups did not find significant differences initially. However, after excluding 25 cases who did not receive any postoperative primary adjuvant therapies (RT or CHT), it was found that patients of WNT subgroup had significantly better OS than patients of other subgroups (SHH subgroup and Non-SHH/WNT subgroup) (**Table 3** **, Figure S2**).

**Table 3:** Univariate analysis of histological and molecular subgroups for OS and EFS in medulloblastoma patients (n = 173).

Factors	No. of cases	5-year OS(%)	p-value	5-year EFS(%)	p-value
Histological subgroups					
CMB	119	51.7	0.187	46.7	0.643
DMB	45	61.0		45.7	
AMB	9	18.5		18.5	
Molecular subgroups					
SHH	40	50.4	0.182	40.9	0.277
WNT	37	69.2		59.2	
Non-SHH/WNT	96	45.9		41.0	
Molecular subgroups^a^					
WNT	30	83.3	**0.028**	78.6	0.081
Other	118	54.9		44.6	

CMB = Classic medulloblastoma, DMB = Desmoplastic/nodular medulloblastoma, AMB = Large-cell/anaplastic medulloblastoma, SHH = SHH pathway medulloblastoma, WNT = WNT pathway medulloblastoma, Non-SHH/WNT =  Non-SHH/WNT pathway medulloblastoma.

a those patients who did not receive any adjuvant therapies after primary resection were excluded.

**Figure 2:**
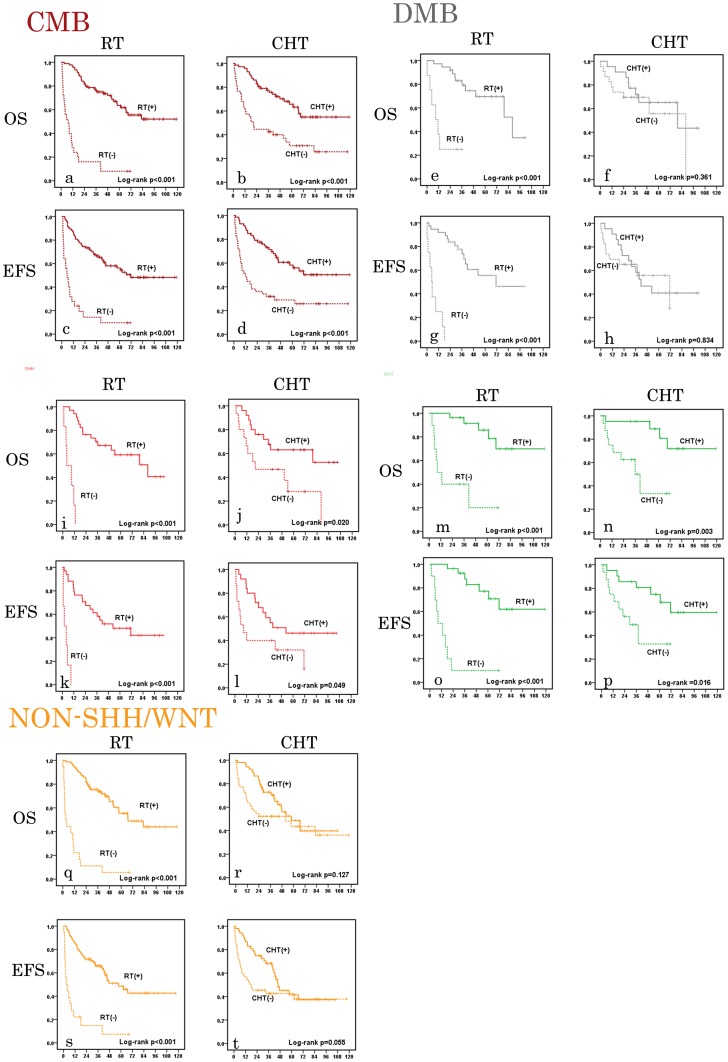
Univariate analysis of overall survival (OS) and event-free survival (EFS) of histological and molecular subgroups of medulloblastoma. a–d: OS and EFS analysis in classic medulloblastoma (CMB, dark red) according to postoperative radiation therapy (RT) and chemotherapy (CHT). e–h: OS and EFS analysis in desmoplastic/nodular medulloblastoma (DMB, grey) according to postoperative RT and CHT. i–l: OS and EFS analysis in SHH medulloblastoma (red) according to postoperative RT and CHT. m–p: OS and EFS analysis in WNT medulloblastoma (green) according to postoperative RT and CHT. q–t: OS and EFS analysis in Non-SHH/WNT medulloblastoma (yellow) according to postoperative RT and CHT. Numbers on the Y axis indicate probability of survival in medulloblastoma patients. Numbers of the X axis indicate the follow-up time (months).

We further studied the influence of postoperative primary adjuvant therapies to the survival of different histological and molecular subgroups of medulloblastoma by univariate analysis. Due to the limited number (9 cases) of AMB, it was not included in the analysis. Postoperative primary RT was found to be a strong prognostic factor influencing the survival in all histological and molecular subgroups. Postoperative primary CHT was found significantly influence the survival of CMB, SHH subgroup and WNT subgroup but not in DMB and Non-SHH/WNT subgroup (**Table S3,** **Figure 2**).

## Discussion

The utility of several prognostic factors, either clinical or pathological, for predicting the clinical outcomes of medulloblastoma patients have been controversially discussed for a long time. Moreover, the sensitivity of different molecular subgroups of MB to current adjuvant therapies is poorly understood. In this study, we analyzed clinical outcomes and prognostic factors in 173 medulloblastoma patients treated at two institutions in China from 2002 to 2011.

There are no infants (age<3 years) in our series. Infants with medulloblastoma usually have a worse outcome compared to older children and adults, mainly due to absence of postoperative RT [15]–[17], so the survivals of our series may be higher than those of the entire group of patients in China. Authors of prospective clinical trials and retrospective studies on medulloblastoma patients mostly considered children (age≥3 years) and adults as different groups of subjects and focused on either one of the two groups. Meanwhile, several reports, including large population-based study, did not find significant difference in survivals between children and adults [8], [17], [18]. The role of sex in predicting the outcomes of medulloblastoma patients is also controversial. There are a few series showing female patients having better prognosis both in children and adults [19]–[21], while others did not find sex influencing treatment outcomes significantly [22]. Our data revealed no independent prognostic significance according to age groups (<16 years vs. ≥16years) and sex in medulloblastoma patients.

The role of two clinical parameters—involvement of brainstem or IV ventricle floor by the tumor at diagnosis and extent of tumor resection, on medulloblastoma patients' outcomes has been contraversial and numerous clinical studies yielded different results [23]–[34]. In the present study, absence of brainstem or IV ventricle floor involvement by the tumor and complete tumor resection significantly correlated with better OS and EFS in our series in the univariate analysis. Nonetheless, these two prognostic factors both lost significance in the multivariate analysis. Possible explanations for this may be that the two factors were intertwined with each other, or that the sample size was not enough to reveal significance. Although the true prognostic value of brainstem or IV ventricle floor involvement and extent of resection are still undetermined, it appears advisable to attain the greatest extent of resection as long as serious surgical complications are avoided.

The use of postoperative RT has provided the greatest improvement in control of tumors and was an integral component of curative treatments of medulloblastoma patients for many years. The commonly used RT dose is 54 Gy to the posterior fossa, and a standard dose around 35 Gy for lesion-free craniospinal target [22], [29], [35]. Notably, there were 34 patients who did not receive postoperative primary RT in our cohort due to various reasons (e.g. refusal or incompliance). The clinical outcomes for these patients were dismal: only 8.0% of the patients survived at the time of 5 years, and most of them had tumor recurrence within the first year after primary resection.

Chemotherapy has been a very important part of treatment for medulloblastoma in the past several years. For children with medulloblastoma, several studies have showed a benefit for the use of CHT in either average-risk or high-risk children with medulloblastoma [7], [22], [34]. Due to the rarity of adult medulloblastoma, there was only one prospective study on treatments of this group until now [36]. The study concluded CHT was helpful in reducing the risk of recurrence and death in high-risk adult patients. However, the role of CHT in average-risk adults with medulloblastoma is still unconfirmed. Our study demonstrated the prognostic significance of CHT for better OS and EFS in children but not in adults. The true value of CHT in adults with medulloblastoma requires further prospective clinical trials.

In recent years, the discovery of WNT subtype in medullolastoma and its relatively favorable outcomes with current therapies have raised the possibility of reducing intensity of adjuvant therapies [6], [9]–[12]. Since our cohort does not include infant patients and WNT subtype was uncommon in this group of patients by previous study [10], the proportion of WNT subtype in our series (21%) is high than other studies which included infants (∼15%) [9], [10]. Initially, patients of WNT subtype did not have a significantly better survival than patients of other molecular subtypes in our series. However, after 25 patients missing primary adjuvant therapies were excluded, a significant survival benefit had been found in patients of WNT subtype than patients with other subtypes. This finding is consistent with previous studies, suggesting the sensitivity of WNT subtype of medulloblastoma to adjuvant therapies and a possibility of reduction in adjuvant therapies. Another important result is that postoperative CHT was found significantly influence the survival of CMB, SHH subgroup and WNT subgroup of MB. Current trend in treating WNT subgroup of MB is to decrease the intensity of therapy [11]. Our finding suggested that WNT subgroup was sensitive to CHT and it would be risky to remove or defer CHT after primary tumor resection in this subgroup. We also found that postoperative primary CHT did not significantly influence the survival of patients with DMB and Non-SHH/WNT medulloblastoma. Rieken et al analyzed 20 patients with DMB and found the addition of postoperative adjuvant CHT did not bring significant survival benefit, like ours [37]. We postulate that the limited sample size of patients with DMB may be the reason and studies of more patients are needed to confirm the role of CHT in DMB. For Non-SHH/WNT medulloblastoma, more recent studies have reclassified this subgroup into Group 3 and Group 4 [10]–[12]. Patients of Group 4 medulloblastoma had an intermediate prognosis as patients of SHH medulloblastoma do, while patients of Group 3 medulloblastoma had the worst outcome [10], [11]. Our data revealed no significant difference between the survivals of patients who received adjuvant CHT and who did not in Non-SHH/WNT subgroup, and this insensitivity to current CHT of the subgroup may be one of the reasons explaining the dismal outcome of Group 3 medulloblastoma. However, further detailed studies are necessary to explore the role of CHT in Non-SHH/WNT medulloblastoma.

One of the limitations of this study is the unavailability of detailed information of postoperative RT and CHT, since detailed protocols of RT and CHT were acquired only in a minority of our cohort. Radiation area/dose and type of chemotherapy are considered as prognostic factors significantly affecting clinical outcome of MB patients [1], [4], [18], [24]. The findings of current study would be strengthened if details of RT and CHT were complete in all the cases and be demonstrated comparable across subgroups of MB. Future studies with more detailed data are needed to further confirm the findings.

In conclusion, we have reported a large series of medulloblastoma patients in China. Our data demonstrated that the administration of adjuvant CHT to children could significantly improve the survival of this group. Moreover, postoperative primary CHT was found significantly influence the survival of CMB, SHH subgroup and WNT subgroup but not in DMB and Non-SHH/WNT subgroup. The role of postoperative CHT in DMB and Non-SHH/WNT medulloblastoma needs further study.

## Supporting Information

Figure S1 **Survival curves of medulloblastoma patients.** a: Overall survival of 173 medulloblastoma patients. **b**: Event-free survival of 173 medulloblastoma patients. **c**: Overall survival of 118 children with medulloblastoma. **d**: Event-free survival of 118 children with medulloblastoma. **e**: Overall survival of 55 adults with medulloblastoma. **d**: Event-free survival of 55 adults with medulloblastoma. Numbers on the Y axis indicate probability of survival in medulloblastoma patients. Numbers of the X axis indicate the follow-up time (months).
(TIF)

Figure S2 **Survival analysis between WNT subgroup (green) and other molecular subgroups (SHH and Non-SHH/WNT subgroups, blue).** a: OS analysis revealed WNT subgroup had significantly better survival than other groups; **b**: EFS analysis revealed WNT subgroup had a trend to significantly better survival than other groups.
(TIF)

Table S1 Immunoreactivity patterns of SHH, WNT, and non-SHH/WNT molecular subgroups*.
(DOC)

Table S2 **Univariate analysis of postoperative adjuvant therapies for OS and EFS in children and adults with medulloblastoma (n = 173).**
(DOC)

Table S3 **Univariate analysis of postoperative adjuvant therapies for OS and EFS in patients with different histological and molecular subgroups of medulloblastoma (n = 173).**
(DOC)
