# Correction: Medulloblastoma in China: Clinicopathologic Analyses of SHH, WNT, and Non-SHH/WNT Molecular Subgroups Reveal Different Therapeutic Responses to Adjuvant Chemotherapy

**DOI:** 10.1371/journal.pone.0105742

**Published:** 2014-08-07

**Authors:** The PLOS ONE Staff

The first three authors, Zhen-Yu Zhang, Jian Xu, and Yong Ren, should be noted as contributing equally to this work.
