# Correction: Medulloblastoma in China: Clinicopathologic Analyses of SHH, WNT, and Non-SHH/WNT Molecular Subgroups Reveal Different Therapeutic Responses to Adjuvant Chemotherapy

**DOI:** 10.1371/journal.pone.0107855

**Published:** 2014-09-08

**Authors:** The PLOS ONE Staff

There are several errors in the published article. The authors are listed out of order. Please view the correct author order, affiliations, and citation here:

Zhen-Yu Zhang^1^, Jian Xu^1^, Yong Ren^2^, Kay Ka-Wai Li^3^, Ho-Keung Ng^3^, Ying Mao^1^, Ping Zhong^1*^, Yu Yao^1*^, Liang-Fu Zhou^1^

1 Department of Neurosurgery, Huashan Hospital, Fudan University, Shanghai, China, 2 Department of Pathology, Wuhan General Hospital of Guangzhou Command, People’s Liberation Army, Wuhan, China, 3 Department of Anatomical and Cellular Pathology, Prince of Wales Hospital, The Chinese University of Hong Kong, Hong Kong, China

Zhang Z-Y, Xu J, Ren Y, Yao Y, Li KK-W, et al. (2014) Medulloblastoma in China: Clinicopathologic Analyses of SHH, WNT, and Non-SHH/WNT Molecular Subgroups Reveal Different Therapeutic Responses to Adjuvant Chemotherapy. PLoS ONE 9(6): e99490. doi:10.1371/journal.pone.0099490

The email address for co-Corresponding Author Yu Yao is incorrect. The correct email address is: yu_yao03@126.com.

Figure 2 is incorrect. The authors have provided a corrected version here.

**Figure 2:**
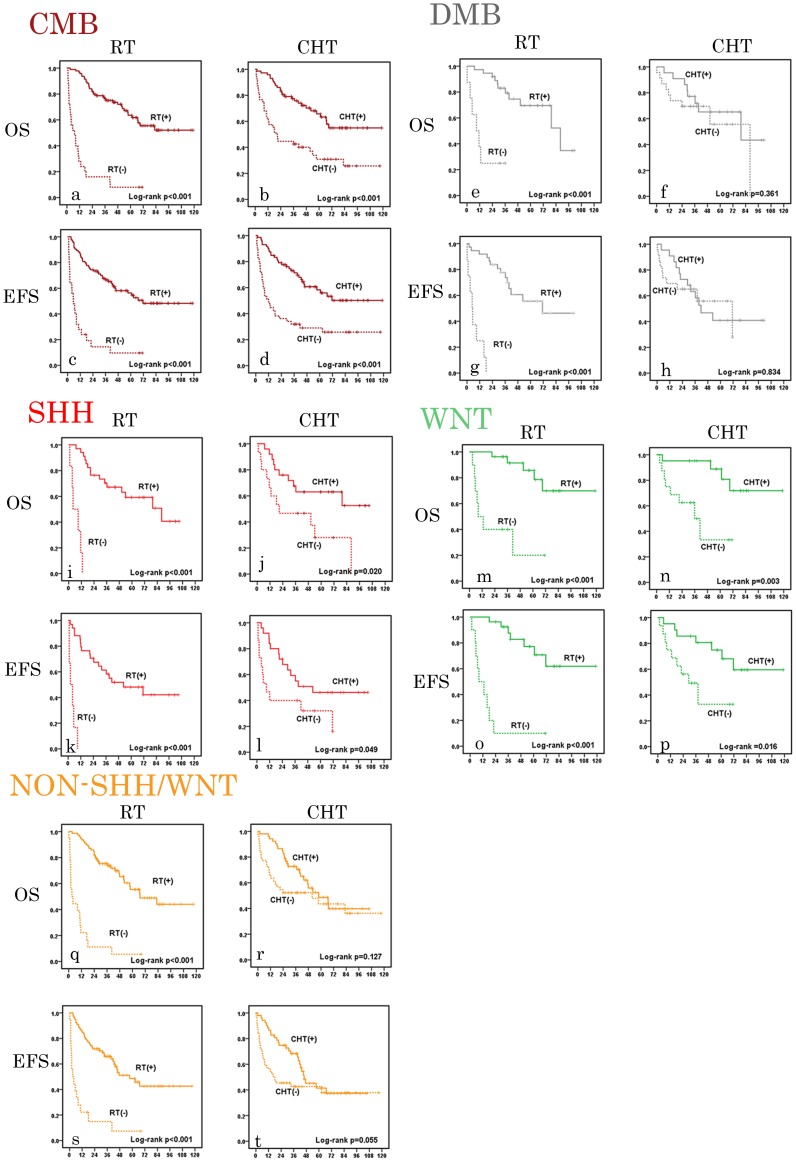
Univariate analysis of overall survival (OS) and event-free survival (EFS) of histological and molecular subgroups of medulloblastoma. a–d: OS and EFS analysis in classic medulloblastoma (CMB, dark red) according to postoperative radiation therapy (RT) and chemotherapy (CHT). e–h: OS and EFS analysis in desmoplastic/nodular medulloblastoma (DMB, grey) according to postoperative RT and CHT. i–l: OS and EFS analysis in SHH medulloblastoma (red) according to postoperative RT and CHT. m–p: OS and EFS analysis in WNT medulloblastoma (green) according to postoperative RT and CHT. q–t: OS and EFS analysis in Non-SHH/WNT medulloblastoma (yellow) according to postoperative RT and CHT. Numbers on the Y axis indicate probability of survival in medulloblastoma patients. Numbers of the X axis indicate the follow-up time (months).
