# Supplementary figures and images for: Medulloblastoma in China: Clinicopathologic Analyses of SHH, WNT, and Non-SHH/WNT Molecular Subgroups Reveal Different Therapeutic Responses to Adjuvant Chemotherapy
Source: PLoS One. 2014 Jun 16;9(6):e99490. doi: 10.1371/journal.pone.0099490 (PMC4059646; doi:10.1371/journal.pone.0099490)

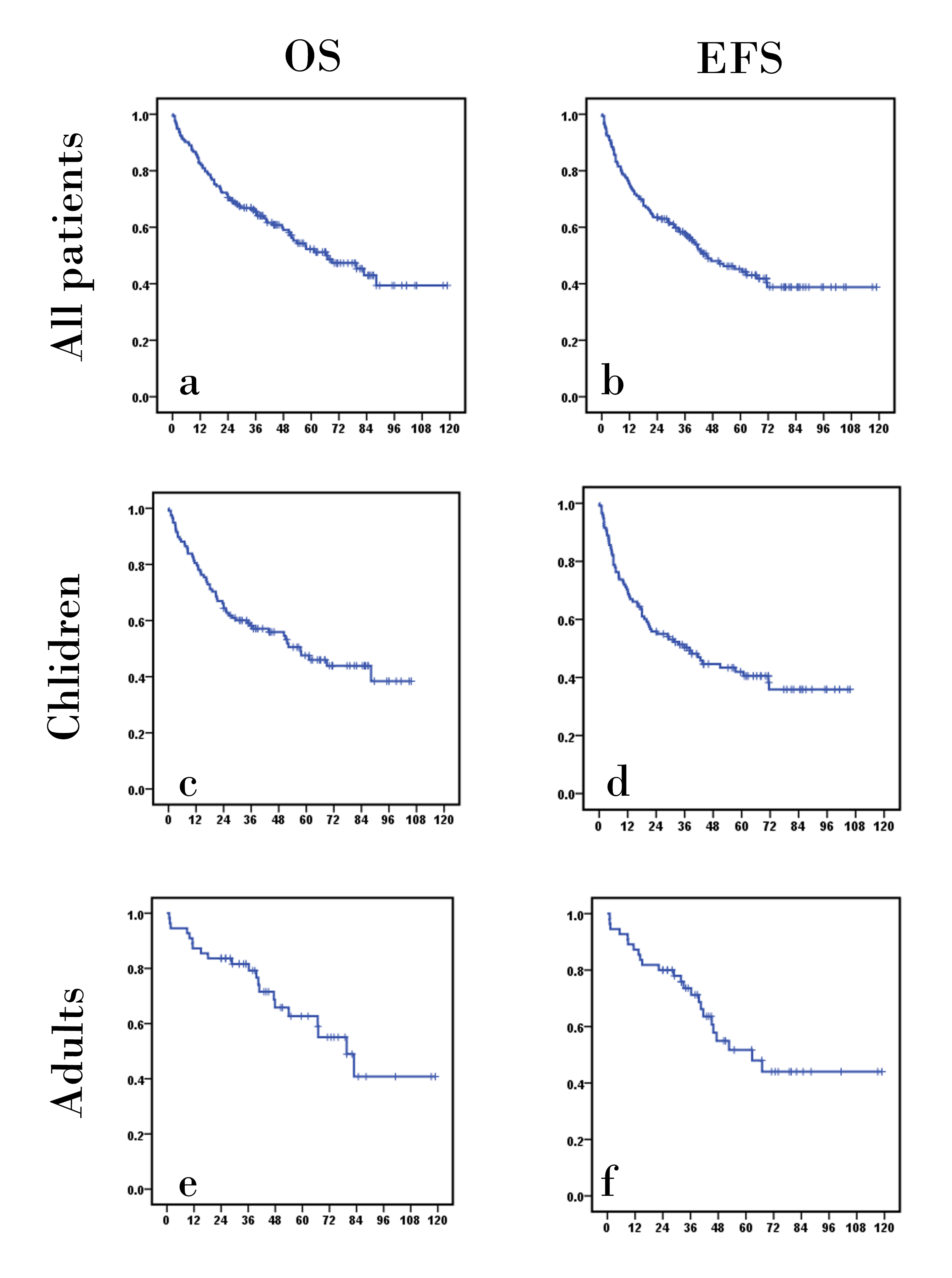

Supplement: Figure S1 — Survival curves of medulloblastoma patients. a: Overall survival of 173 medulloblastoma patients. b: Event-free survival of 173 medulloblastoma patients. c: Overall survival of 118 children with medulloblastoma. d: Event-free survival of 118 children with medulloblastoma. e: Overall survival of 55 adults with medulloblastoma. d: Event-free survival of 55 adults with medulloblastoma. Numbers on the Y axis indicate probability of survival in medulloblastoma patients. Numbers of the X axis indicate the follow-up time (months). (TIF) [file pone.0099490.s001.tif]

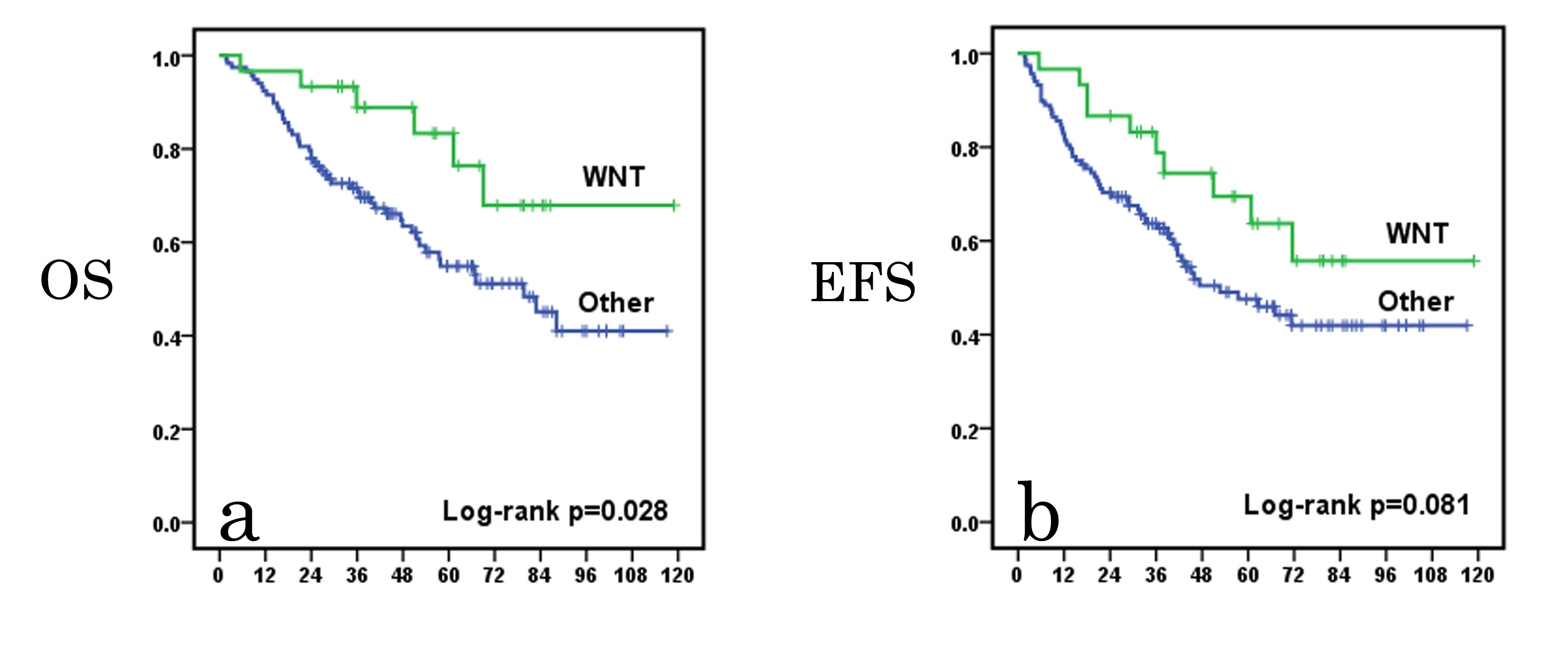

Supplement: Figure S2 — Survival analysis between WNT subgroup (green) and other molecular subgroups (SHH and Non-SHH/WNT subgroups, blue). a: OS analysis revealed WNT subgroup had significantly better survival than other groups; b: EFS analysis revealed WNT subgroup had a trend to significantly better survival than other groups. (TIF) [file pone.0099490.s002.tif]
