# Supplementary material for: Medulloblastoma in China: Clinicopathologic Analyses of SHH, WNT, and Non-SHH/WNT Molecular Subgroups Reveal Different Therapeutic Responses to Adjuvant Chemotherapy
Source: PLoS One. 2014 Jun 16;9(6):e99490. doi: 10.1371/journal.pone.0099490 (PMC4059646; doi:10.1371/journal.pone.0099490)
Supplement: Table S1 — Immunoreactivity patterns of SHH, WNT, and non-SHH/WNT molecular subgroups*. (DOC) [file pone.0099490.s003.doc]

**Table S1** Immunoreactivity patterns of SHH, WNT, and non-SHH/WNT molecular subgroups*

| Molecular subgroup | Immunoreactivity patterns | |
| --- | --- | --- |
|  | GAB1 | β-Catenin |
| SHH | Cytoplasmic | Cytoplasmic |
| WNT | Negative | Nuclear+cytoplasmic |
| Non-SHH/WNT | Negative | Cytoplasmic |

* Ellison et al. [11]
