# Supplementary material for: Medulloblastoma in China: Clinicopathologic Analyses of SHH, WNT, and Non-SHH/WNT Molecular Subgroups Reveal Different Therapeutic Responses to Adjuvant Chemotherapy
Source: PLoS One. 2014 Jun 16;9(6):e99490. doi: 10.1371/journal.pone.0099490 (PMC4059646; doi:10.1371/journal.pone.0099490)
Supplement: Table S2 — Univariate analysis of postoperative adjuvant therapies for OS and EFS in children and adults with medulloblastoma (n = 173). (DOC) [file pone.0099490.s004.doc]

**Table S2** Univariate analysis of postoperative adjuvant therapies for OS and EFS in children and adults with medulloblastoma (n=173)

| Subgroups | No. of cases | 5-year OS(%) | p-value | 5-year EFS(%) | p-value |
| --- | --- | --- | --- | --- | --- |
| Children | 118 |  |  |  |  |
| RT |  |  |  |  |  |
| Yes | 92 | 58.0 | **＜0.001** | 51.9 | **＜0.001** |
| No | 26 | 11.5 |  | 5.1 |  |
| CHT |  |  |  |  |  |
| Yes | 63 | 58.8 | **0.001** | 49.3 | **0.003** |
| No | 55 | 33.4 |  | 35.1 |  |
| Adults | 55 |  |  |  |  |
| RT |  |  |  |  |  |
| Yes | 47 | 72.4 | **＜0.001** | 58.6 | **＜0.001** |
| No | 8 | 12.5 |  | 12.5 |  |
| CHT |  |  |  |  |  |
| Yes | 35 | 68.5 | 0.389 | 53.7 | 0.165 |
| No | 20 | 53.3 |  | 51.4 |  |

RT= postoperative primary radiation therapy, CHT= postoperative primary chemotherapy
