# Supplementary material for: Medulloblastoma in China: Clinicopathologic Analyses of SHH, WNT, and Non-SHH/WNT Molecular Subgroups Reveal Different Therapeutic Responses to Adjuvant Chemotherapy
Source: PLoS One. 2014 Jun 16;9(6):e99490. doi: 10.1371/journal.pone.0099490 (PMC4059646; doi:10.1371/journal.pone.0099490)
Supplement: Table S3 — Univariate analysis of postoperative adjuvant therapies for OS and EFS in patients with different histological and molecular subgroups of medulloblastoma (n = 173). (DOC) [file pone.0099490.s005.doc]

**Table S3** Univariate analysis of postoperative adjuvant therapies for OS and EFS in patients with different histological and molecular subgroups of medulloblastoma (n=173)

| Subgroups | No. of cases | 5-year OS(%) | p-value | 5-year EFS(%) | p-value |
| --- | --- | --- | --- | --- | --- |
| CMB | 119 |  |  |  |  |
| RT |  |  |  |  |  |
| Yes | 94 | 63.6 | **＜0.001** | 56.3 | **＜0.001** |
| No | 25 | 8.0 |  | 9.6 |  |
| CHT |  |  |  |  |  |
| Yes | 72 | 65.7 | **＜0.001** | 58.3 | **＜0.001** |
| No | 47 | 30.8 |  | 29.0 |  |
| DMB | 45 |  |  |  |  |
| RT |  |  |  |  |  |
| Yes | 37 | 69.6 | **＜0.001** | 50.0 | **＜0.001** |
| No | 8 | 25.0 |  | 0.0 |  |
| CHT |  |  |  |  |  |
| Yes | 22 | 65.2 | 0.361 | 40.9 | 0.834 |
| No | 23 | 55.7 |  | 55.9 |  |
| SHH | 40 |  |  |  |  |
| RT |  |  |  |  |  |
| Yes | 34 | 59.3 | **＜0.001** | 52.7 | **＜0.001** |
| No | 6 | 0.0 |  | 0.0 |  |
| CHT |  |  |  |  |  |
| Yes | 25 | 63.1 | **0.020** | 46.2 | **0.049** |
| No | 15 | 28.0 |  | 32.0 |  |
| WNT | 37 |  |  |  |  |
| RT |  |  |  |  |  |
| Yes | 27 | 85.8 | **＜0.001** | 77.2 | **＜0.001** |
| No | 10 | 20.0 |  | 10.0 |  |
| CHT |  |  |  |  |  |
| Yes | 21 | 88.9 | **0.003** | 74.9 | **0.016** |
| No | 16 | 33.3 |  | 32.8 |  |
| Non-SHH/WNT | 96 |  |  |  |  |
| RT |  |  |  |  |  |
| Yes | 78 | 55.4 | **＜0.001** | 43.2 | **＜0.001** |
| No | 18 | 5.6 |  | 8.3 |  |
| CHT |  |  |  |  |  |
| Yes | 52 | 48.7 | 0.127 | 41.4 | 0.055 |
| No | 44 | 43.6 |  | 42.6 |  |

CMB=Classic medulloblastoma, DMB=Desmoplastic/nodular medulloblastoma, SHH=SHH pathway medulloblastoma, WNT=WNT pathway medulloblastoma, Non-SHH/WNT= Non-SHH/WNT pathway medulloblastoma, RT= postoperative primary radiation therapy, CHT= postoperative primary chemotherapy
